# Local injection therapy for carpal tunnel syndrome: a network meta-analysis of randomized controlled trial

**DOI:** 10.3389/fphar.2023.1140410

**Published:** 2023-08-24

**Authors:** TianQi Zhou, ZhuoRao Wu, XingYun Gou, HaiSha Xia, JiLin Ding, ShuangChun Ai

**Affiliations:** ^1^ College of Health and Rehabilitation, Chengdu University of Traditional Chinese Medicine, Chengdu, China; ^2^ Department of Rehabilitation Medicine, Mianyang Hospital, Chengdu University of Traditional Chinese Medicine, Mianyang, China

**Keywords:** carpal tunnel syndrome, local injection therapy, platelet-rich plasma, 5% dextrose in water, clinical efficacy, network meta-analysis

## Abstract

**Objective:** Clinical research has shown that local injections for carpal tunnel syndrome reduce the symptoms of patients and enhance their quality of life considerably. However, there are several therapy options, and the optimal choice of regimen remains uncertain. Therefore, we comprehensively evaluated the variations in clinical efficacy and safety of several medications for treating carpal tunnel syndrome.

**Methods:** Computer searches of Embase, PubMed, Cochrane Library, and Web of Science databases were used to collect articles of randomized controlled trials on local injections for treating carpal tunnel syndrome from database creation till 10 June 2023. Two researchers independently screened the literature, extracted information, evaluated the risk of bias in the included studies, and performed network Meta-analysis using Stata 17.0 software. Drug efficacy was assessed using symptom severity/function and pain intensity. Surface under the cumulative ranking curve (SUCRA) ranking was used to determine the advantage of each therapy.

**Results:** We included 26 randomized controlled trials with 1896 wrists involving 12 interventions, such as local injections of corticosteroids, platelet-rich plasma, 5% dextrose, progesterone, and hyaluronidase. The results of the network meta-analysis showed the following: (i) symptom severity: at the 3-month follow-up, D5W combined with splinting (SUCRA = 95%) ranked first, and hyaluronidase (SUCRA = 89.6%) at 6 months; (ii) functional severity: either at the 3-month follow-up (SUCRA = 89.5%) or 6 months (SUCRA = 83.6%), iii) pain intensity: 5% dextrose in water combined with splinting was the most effective at the 3-month (SUCRA = 85%) and 6-month (SUCRA = 87.6%) follow-up.

**Conclusion:** Considering the combination of symptoms/function and pain intensity, combining 5% dextrose in water with splinting is probably the treatment of choice for patients with carpal tunnel syndrome. It is more effective than glucocorticoids and no adverse effects have been observed.

**Systematic Review Registration:** https://www.crd.york.ac.uk/PROSPERO/, identifier CRD42022370525.

## 1 Introduction

Carpal tunnel syndrome (CTS) is a commonly observed nerve entrapment disorder characterized by numbness and pain in the area of median nerve innervation, which may radiate in all directions and involve the shoulder and elbow (Katz and Simmons, 2002; Hesami et al., 2018). The prevalence of CTS in the general population is as high as 14.3%, and the prevalence of electrophysiologically diagnosed diseases ranges is from ∼2.7 to 4.9% (Atroshi et al., 1999). CTS occurs in all age groups, but a majority of cases occur in individuals aged from 40 to 60 years; the ratio of men to women with CTS is 3:7 (Kanaan and Sawaya, 2001). The Italian National Insurance Institute for Accidents at Work calculated that CTS accounted for 57% of all work-related musculoskeletal disorders in 2000 (Alfonso et al., 2010). In the United States, 3 of every 10,000 full-time workers exhibit CTS (Occupationaldiseases et al., 2004). Temporary or permanent disabilities to CTS are increasing, as are the associated medical costs, thereby imposing a substantial financial burden on families and society.

The current management of CTS is mainly conservative and surgical release (Graham et al., 2016; Padua et al., 2016). Conventional treatment is used for mild to moderate cases, and surgical release is considered after conservative treatment has failed. Traditional treatment options commonly include topical corticosteroid (CS) injections and neutral splinting (Padua et al., 2016). Studies have reported that topical CS injections are effective in the short term but not in the long term and may cause local erythema, crystal-induced synovitis, and hyperthermia (Marshall et al., 2007). Splinting has a slow onset of action and provides less pain relief compared with local injections do (vanVeen et al., 2015; Raeissadat et al., 2018). Thus, a simple, effective, and durable treatment option is urgently necessary.

The use of several emerging topical, injectable agents as options for treating CTS has been attempted. Of them, platelet-rich plasma (PRP) and 5% dextrose in water (D5W) are the most critical potential treatments (Lin et al., 2020; Hong et al., 2022). Ozone O) and hyaluronidase (HA) have also shown potential (Elawamy et al., 2020; Forogh et al., 2021). Progesterone and insulin have also shown efficacy in the treatment of CTS in women and diabetics that is not inferior to classical treatment regimens (corticosteroids and splints) (Bahrami et al., 2015; Kamel et al., 2019). In a network meta-analysis comparing PRP, D5W, CS, splints, and saline, PRP and D5W were superior to CS in improving symptom severity and functional status (Lin C. P. et al., 2020). Pan et al. further compared the efficacy of PRP, D5W, progesterone, ozone, and various types and doses of CS by using the same approach as that described by Lin et al., and the results of the former were consistent with those of the latter (Hong et al., 2022). However, the aforementioned studies are deficient. They did not consider local injection therapy combined with splinting as a separate intervention but as the same intervention as local injection therapy alone; In addition, the interventions used were not comprehensive. Therefore, we conducted a more comprehensive search relative to the two previous MeSH meta-analyses, including different local injection treatment options and combining local injection treatment with splinting as a separate intervention. Moreover, a network meta-analysis (NMA) was re-run to determine the effectiveness of different local drug injections for treating patients with CTS and to provide a reference for the clinical management of CTS.

## 2 Methods

This systematic review and meta-analysis were conducted in accordance with A Measurement Tool to Assess Systematic Reviews and reported according to the Preferred Reporting Items for Systematic Reviews and Meta-Analyses guidelines (Page et al., 2021). Go to the International Systematic Review Registration Platform website to enroll in the study and receive the registration number: CRD42022370525.

### 2.1 Database and search

Computer searches of the PubMed, Embase, The Cochrane Library, and Web of Science databases were performed to collect randomized controlled trials on local injections of corticosteroids, PRP, 5% dextrose in water, progesterone, ozone, hyaluronic acid, and splints for the treatment of CTS. The search period was from the creation of the database until 10 June 2023. The search was conducted using a combination of MeSH and free terms adapted to the characteristics of each database. References to relevant subject meta-analyses and gray literature were also searched to supplement access to pertinent information. The keywords used in the search were carpal tunnel syndromes, steroids, hyaluronidase, 5% dextrose in water, PRP, splints, ozone, progesterone, and randomized controlled trial. The specific search formula is presented in Supplementary Table S1.

### 2.2 Inclusion and exclusion criteria

Inclusion criteria were the following.

1) Participants were patients older than 18 years who fulfilled the criteria for the CTS clinical and electrophysiological diagnosis.
2) Interventions were local steroids, PRP, glucose, progesterone, hyaluronic acid, or ozone for the experimental group and corticosteroids, saline, local anesthetics, and splints for the control group.
3) The Boston Carpal Tunnel Questionnaire (BCTQ), Symptom Severity Scale (SSS), Functional Severity Scale (FSS), and visual analog score (VAS) were the end measures (Levine et al., 1993), and all the studies had to contain at least one of these markers.
4) Randomized controlled trial
5) English language

Exclusion criteria were animal studies, observational studies, conference abstracts, case reports, reviews, systematic reviews, or literature with incomplete data.

### 2.3 Study selection

The downloaded literature data were searched for duplicate title information, using EndNote X9 software. Two researchers (Zhou/Wu) independently screened the literature, extracted information, and cross-checked it. Disagreements were resolved through discussion or negotiation with a third party. The literature was screened by first reading the article’s title, and after eliminating irrelevant documents, the abstract and full text was further read to determine whether the articles could be included. Contact the original study authors by email or telephone to obtain missing data.

### 2.4 Data extraction

Two researchers (Zhou/Wu) independently extracted and cross-checked the following information: 1) basic information on the included studies: first author, time of publication, diagnostic criteria, positioning method, and study type; 2) baseline characteristics (age, sex, sample size, follow-up time, severity) and interventions of the study population; 3) key elements of risk of bias evaluation; and 4) outcome indicators and outcome measures of interest (SSS, FSS, VAS). All the continuous variables were combined for different dose subgroups according to the Cochrane Handbook; the means and standard deviations of change were calculated by means and standard deviations at baseline and endpoints (Higgins et al., 2011). All extracted outcome indicators were from the March and June follow-ups, or if the follow-up was less than 3 months, the data were collected as close to March as possible. Disagreements were resolved through discussion or third-party negotiation.

### 2.5 Risk of bias assessment

The quality of each study was assessed independently by two investigators (Zhou/Wu). The quality of the randomized controlled trials was evaluated using the Cochrane Risk-Of Bias tool 2.0. The tool included six domains used for methodological assessment: 1) random sequence generation, 2) allocation concealment, 3) blinding of participants and personnel, 4) blinding of outcome assessment, 5) incomplete outcome data, and 6) selective reporting (Cumpston et al., 2019). Each study was categorized as low risk, high risk, or unclear risk, and disagreements were resolved through discussion or consultation with a third party.

### 2.6 Statistical analysis

Stata 17.0 software was used for statistical analysis. The outcome indicators were continuous variables, and the unit measures of the outcome indicators were the same between studies; thus, mean difference (MD) and 95% confidence interval (CI) were used as effect measures. The NMA was conducted using the “network” package of Stata 17.0 software, and the network relationships were mapped out. An inconsistency model was used to test the overall inconsistency. If the difference was not statistically significant (*p > 0*.05), the inconsistency did not exist. The local inconsistency test then used the “node splitting method.” If the difference between the split points was not statistically significant (*p > 0*.05), the consistency model was used for analysis. If the relationship graph contained a closed loop, the loop inconsistency (LF) factor was used to determine whether there was an inconsistency. If the lower 95% CI limit for the inconsistency factors’ (IFs’) value was 0 or nearly 0, the direct and indirect evidence were considered consistent. If there was a closed loop in the relationship diagram, its inconsistency was judged by the loop inconsistency factor. If the lower limit of 95% CI IFs’ value was 0 or close to 0, the direct and indirect evidence was consistent (Chao et al., 2014). Finally, comparing the cumulative area under the cumulative ranking curve (SUCRA) of the probability plot describes the ranking between interventions (Salanti et al., 2011). As the value of SUCRA increases, the effectiveness of the intervention increases. In addition, comparison-corrected funnel plots were created using Stata17 to verify the presence of bias between studies (Macaskill et al., 2001).

## 3 Results

### 3.1 Study selection

**FIGURE 1:**
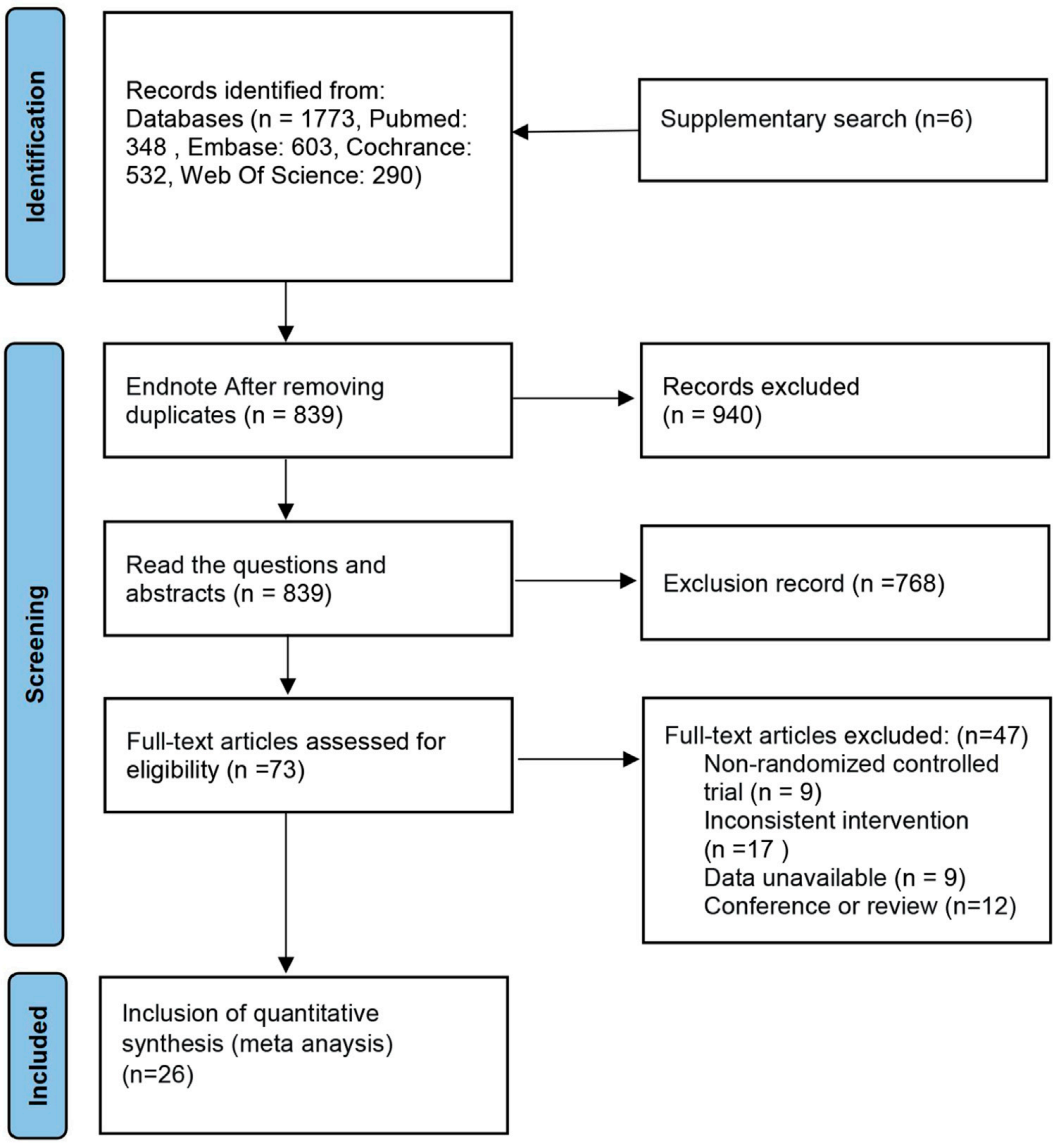
Flowchart of selection of included studies.

The database search obtained 1,594 articles. EndNote X9 software eliminated duplicate literature (866 articles). Of the remaining 728 articles, after reading the title and abstract, 668 articles were excluded for not fulfilling the inclusion criteria. The remaining 60 articles were used in the full-text re-screening. The final sample had 26 articles, with a total of 1896 wrists. The process is shown in Figure 1. Of the included literature, five were three-arm trials (Karadaş et al., 2011; Karadaş et al., 2012; Atroshi et al., 2013; Hesami et al., 2018; Salman Roghani et al., 2018), and the rest were two-arm trials. Thirteen studies used ultrasound-guided injections (Wu et al., 2017a; Wu et al., 2017b; Wang et al., 2017; Salman Roghani et al., 2018; Wu et al., 2018; Alsaeid, 2019; Senna et al., 2019; Shen et al., 2019; Elawamy et al., 2020; Chen et al., 2021; Forogh et al., 2021; Su et al., 2021; Babaei-Ghazani et al., 2022). Four papers did not specify the severity of patients’ symptoms (Peters-Veluthamaningal et al., 2010; Karadaş et al., 2011; Karadaş et al., 2012; So et al., 2018), one was moderately severe (Su et al., 2021), and the rest were mild to moderate. The literature included in this paper relates to treatment with CS, PRP, 5% dextrose (D5W), progesterone P), hyaluronidase (HA), ozone O), local anesthetics, (LA), saline (NSS) and splinting S), and the severity of patient’s symptoms was mainly mild to moderate. The details are shown in Table 1.

**TABLE 1:** Characteristics of included studies.

Year	Author	Diagnosis	n (F/M)	Age (SD)	Treatment	US(Y/N)	Outcome	Follow-up (month)	Degree
2010	Peters-Veluthamaningal	EDS + clinical	26/7	56.5 ± 15.14	CS:Triamcinolone acetonide 1 mL	N-US	①②	12	NA
			27/9	57.60 ± 40.30	NSS:Normal saline 1 mL				
2020	Hashim	EDS + clinical	17/3	48.8 ± 7.45	PRP1:Lidocaine 0.5 mL + Platelet-rich plasma 1 mL	N-US	①②③	3	Mild to moderate
			18/2	48.8 ± 6.62	PRP2:Lidocaine 0.5 mL + Platelet-rich plasma 1 mL				
			18/2	49.15 ± 6.06	CS: Lidocaine 0.5 mL + Methylprednisolone 40 mg				
2020	Elawamy	EDS + clinical	17/13	40.7 ± 6.5	HA:Hyaluronidase + NSS 10 mL	Y-US	①②③	6	Mild to moderate
			17/13	38.3 ± 5.4	NSS:Normal saline 10 mL				
2021	Yu-Chi	EDS + clinical	13/4	50.9 ± 2.5	HA:Hyaluronidase 25 mg/2.5 mL	Y-US	①②③	6	Mild to moderate
			12/3	58.9 ± 2.8	NSS:Normal saline 2.5 mL				
2022	Babaei-Ghazani	EDS + clinical	27	48.63 ± 11.88	D5W:5% dextrose 5 mL	Y-US	①②③	6	Mild to moderate
			27	49.33 ± 8.73	CS: Triamcinolone (40 mg/mL) 1 mL				
2019	Alsaeid	EDS + clinical	11/9	40.18 ± 10.5	CS: Dexamethasone (4 mg/mL) 2 mL + 0.5% Bupivacaine 3 mL	Y-US	①②	6	Mild to moderate
			10/10	42.76 ± 8.3	HA: Hyaluronidase 300 IU in NSS 2 mL + 0.5% Bupivacaine 3 mL				
2021	Si-Ru	EDS + clinical	21/3	53 ± 2.0	PRP: Platelet-rich plasma 3.5 mL	Y-US	①②	12	Moderate to severe
					NSS:Normal saline 3.5 mL				
2019	Shen	EDS + clinical	25/1	56.8 ± 1.7	PRP: Platelet-rich plasma 3 mL	Y-US	①②	6	Moderate
			22/4	58.5 ± 2.1	D5W:5% dextrose 3 mL				
2019	Senna	EDS + clinical	35/8	38.3 ± 6.4	PRP:Platelet-rich plasma 2 mL	Y-US	①②③	3	Mild to moderate
			36/6	40.7 ± 9.4	CS: Methylprednisolone (40 mg/mL) 1 mL				
2018	Wu	EDS + clinical	22/5	58.6 ± 2.2	D5W:5% dextrose 5 mL	Y-US	①②③	6	Mild to moderate
			21/6	54.3 ± 2.0	CS: Triamcinolone (10 mg/mL) 3 mL + Normal saline 2 mL				
2013	Atroshi	EDS + clinical	26/11	47 ± 12	CS1:Methylprednisolone 2 mL (80 mg) + Lidocaine 1 mL	N-US	①③	6	Mild to moderate
			27/10	44 ± 11	CS2:Methylprednisolone 1 mL (40 mg) + Normal saline 1 mL + Lidocaine 1 mL				
			28/9	49 ± 11	NSS:Normal saline 2 mL + Lidocaine 1 mL				
2012	Karadaş	EDS + clinical	17/3	46.40 ± 11.60	CS: Triamcinolone 40 mg 1 mL	N-US	①②③	6	NA
			16/2	46.83 ± 5.97	LA:1% Procaine 4 mL				
			17/2	48.40 ± 12.13	NSS:Normal saline 1 mL				
2011	Karadaz	EDS + clinical	29/5	48.02 ± 12.58	CS:Triamcinolone acetonide 40 mg	N-US	③	6	NA
			28/4	46.75 ± 5.83	LA:1% Procaine 4 mL				
			29/4	46.35 ± 12.38	CS: Triamcinolone acetonide 40 mg + 1% Procaine 4 mL				
2017	Raeissadat	EDS + clinical	41/0	51	CS:2% Lidocaine 0.5 mL + Triamcinolone (40 mg/mL) 0.5 mL	N-US	①②③	6	Mild to moderate
			37/0	47	P:2% Lidocaine 0.5 mL + Hydroxy progesterone (500 mg/2 mL 0.5 mL				
2017	Wu	EDS + clinical	27/3	57.87 ± 1.51	PRP: Platelet-rich plasma 3 mL	Y-US	①②③	6	Mild to moderate
			25/5	54.27 ± 1.34	S: Splint				
2015	Bahrami	EDS + clinical	30/0	51.7 ± 9.7	CS: Triamcinolone acetate (40 mg/mL) 0.5 mL + 2% Lidocaine 0.5 mL	N-US	①②③	2.5	Mild to moderate
			24/0	48.2 ± 9.8	P:2% Lidocaine 0.5 mL + Hydroxy progesterone (500 mg/2 mL 0.5 mL				
2022	Burton	EDS + clinical	100		CS:DepoMedrone (40 mg/mL) 20 mg	N-US	②③	12	Mild to moderate
			105		S: Splint				
2017	Ho SO	EDS + clinical	21/4	57.32 ± 9.12	CS:20 mg methylprednisolone + lidnocaine	N-US	①②	1	NA
			22/3	57.28 ± 9.75	S: Splint				
2018	Chesterton	EDS + clinical	73/43	52.6 ± 17.0	CS: Methylprednisolone acetate (40 mg/mL) 20 mg	N-US	①②	6	Mild to moderate
			81/37	52.2 ± 14.9	S: Splint				
2017	Wu	EDS + clinical	26/4	58.47 ± 2.33	D5W:5% dextrose 5 mL	Y-US	①②③	6	Mild to moderate
			24/6	58.10 ± 1.93	NSS:Normal saline 5 mL				
2020	Forough	EDS + clinical	18	54.70 ± 6.60	0 + S: Ozone 3 mL + Lidocaine 10 μg/mL 1 mL + Splint	Y-US	①②③	3	Mild to moderate
			18	53.65 ± 9.26	CS + S:Triamcinolone 40 mg + Lidocaine 1 mL + Splint				
2019	Bahrami	EDS + clinical	18	48.27 ± 3.33	: Ozone 4 mL (10 mg/dL) + 1 mL Lidocaine + Splint	N-US	①②③	2.5	Mild to moderate
			20	46.35 ± 6.3	S: Splint				
2018	Raeissadat	EDS + clinical	21	51.20 ± 9.82	PRP + S:Platelet-rich plasma 1 mL + 0.5 mL Lidocaine + Splint	N-US	①②③	2.5	Mild
			20	47.23 ± 7.11	S: Splint				
2018	Roghani	EDS + clinical	22/10	66.1 ± 13.4	CS1+S: Triamcinolone (40 mg/mL) 2 mL + 2% Lidocaine 1 mL + splint	Y-US	①②③	6	Moderate
			28/4	66 ± 10	CS2+S: Triamcinolone (40 mg/mL) 1 mL + 2% Lidocaine 2 mL + splint				
			27/3	63.4 ± 10.7	S:2% Lidocaine + Normal saline 3 mL + splint				
2022	Aghaei	EDS + clinical	17/3	52.3 ± 6.7	D5W + S:5% dextrose 2 mL + splint	N-US	①②③	12	Mild to moderate
			17/3	49.6 ± 9.0	CS + S: Triamcinolone (40 mg/mL) 1 mL + Normal saline 1 mL + splint				
2017	Wang	EDS + clinical	20/6	54.34 ± 9.86	CS + S:10 mg (10 mg/mL) triamcinolone +2% lidocaine hydrochloride + splint	Y-US	①②③	3	Mild to moderate
			21/5	55.76 ± 8.56	CS:10 mg (10 mg/mL) triamcinolone +2% lidocaine hydrochloride				

EDS, electrophysiological diagnosis; CS, corticosteroids; PRP, platelet-rich plasma; D5W, 5% dextrose in water; P, progesterone; HA, hyaluronidase; O, ozone; LA, local anesthetic; NSS, normal saline; S, splint; “+”, combine; US, ultrasound; NA, not reported; ① VAS, visual analog scale; ② SSS, symptom severity score; ③ FSS, functional status scale.

### 3.2 Risk of bias evaluation results

**FIGURE 2:**
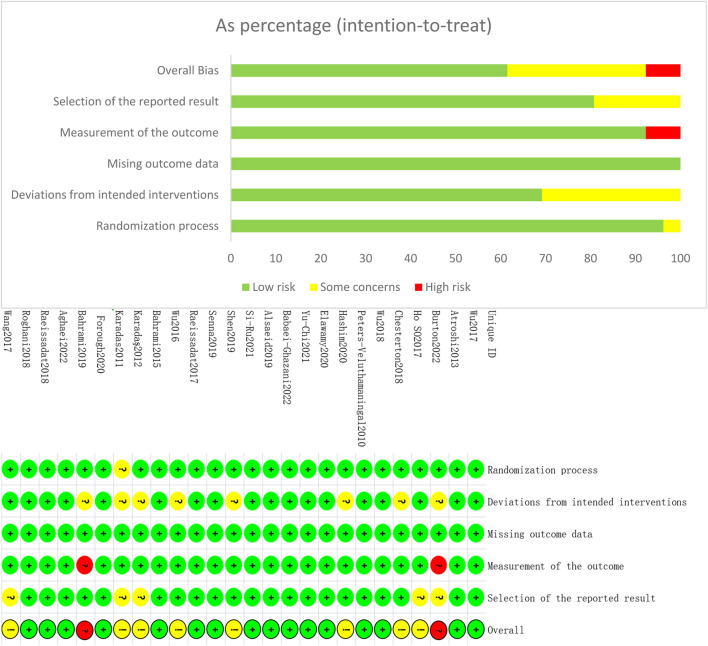
Risk of bias summary. Risk of bias graph.

Six studies did not describe specific randomization methods (Karadaş et al., 2011; Karadaş et al., 2012; Raeissadat et al., 2017; Alsaeid, 2019; Elawamy et al., 2020; Forogh et al., 2021). Thirteen studies described allocation concealment (Peters-Veluthamaningal et al., 2010; Atroshi et al., 2013; Bahrami et al., 2015; Wu et al., 2017b; Raeissadat et al., 2017; Salman Roghani et al., 2018; Senna et al., 2019; Elawamy et al., 2020; Chen et al., 2021; Forogh et al., 2021; Su et al., 2021; Aghaei et al., 2022; Babaei-Ghazani et al., 2022). All participants in the 10 studies completed follow-up (Wu et al., 2017a; Wang et al., 2017; Raeissadat et al., 2018; Wu et al., 2018; Alsaeid, 2019; Shen et al., 2019; Elawamy et al., 2020; Hashim et al., 2020; Forogh et al., 2021; Aghaei et al., 2022). Thirteen studies stated the reasons for missing visits (Peters-Veluthamaningal et al., 2010; Atroshi et al., 2013; Bahrami et al., 2015; Raeissadat et al., 2017; Wu et al., 2017b; Chesterton et al., 2018; Salman Roghani et al., 2018; Bahrami et al., 2019; Senna et al., 2019; Chen et al., 2021; Su et al., 2021; Babaei-Ghazani et al., 2022; !!! INVALID CITATION !!!), and three studies did not (Karadaş et al., 2011; Karadaş et al., 2012; So et al., 2018). Two studies were not blinded to the outcome evaluators because their outcome indicators were subjective scales that may have influenced outcome judgments and were assessed as high risk (Bahrami et al., 2019; Burton et al., 2023). Overall, the quality of the included literature was high, and the risk of bias was low. The results were plotted using ROB2.0 and are shown in Figure 2.

### 3.3 NMA results

#### 3.3.1 Network evidence map between the interventions

The network relationship between various treatment measures is presented in Figure 33: the letters represent the corresponding intervention, the circle size denotes the number of individuals using this intervention, and the thickness of the lines between the different letters represents the number of studies.

#### 3.3.2 Effect of symptom severity score (3-month follow-up)

**FIGURE 3:**
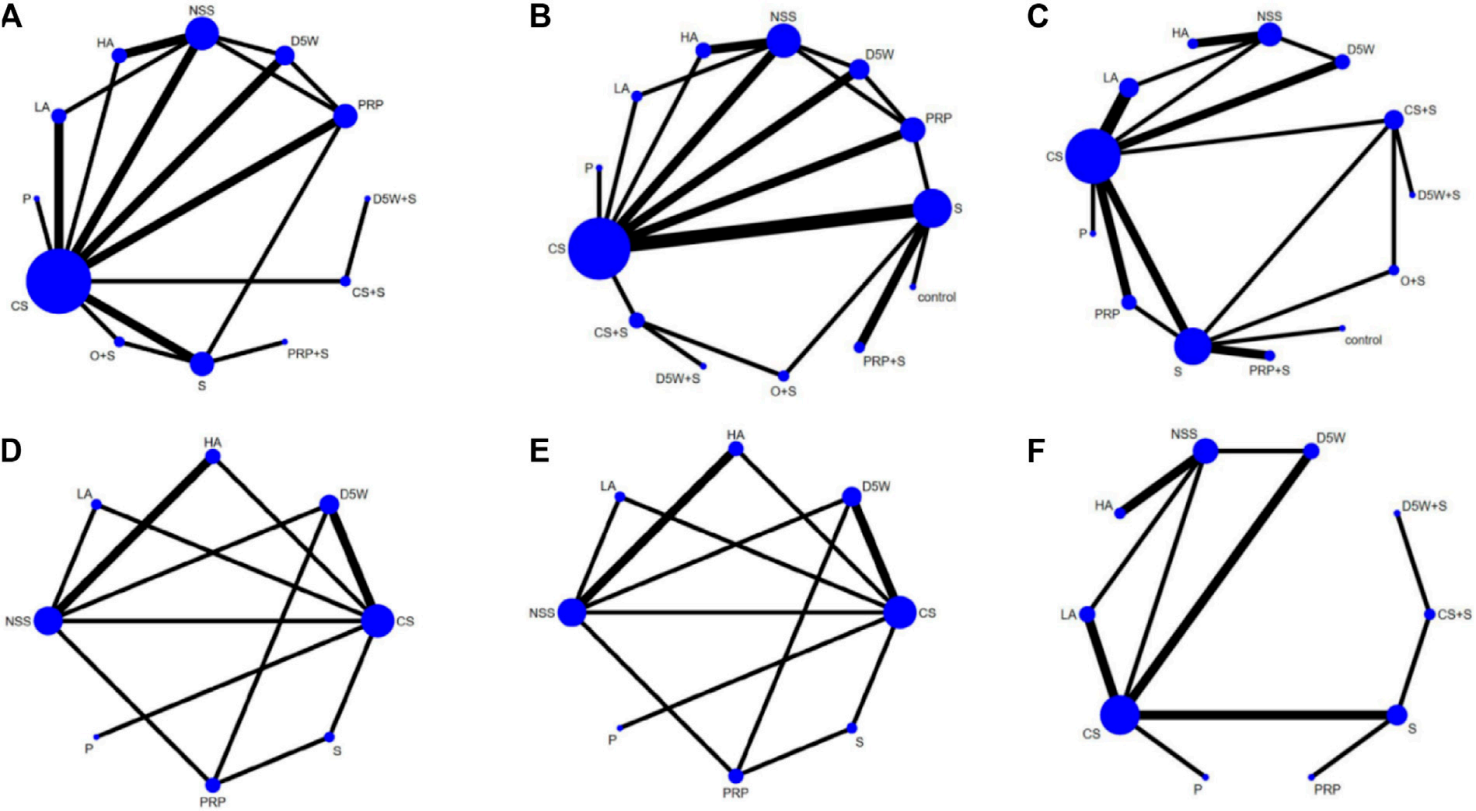
Network plots at 3-month follow-up, **(A)** (SSS), **(B)** (FSS), **(C)** (VAS); At 6-month follow-up, **(D)** (SSS), **(E)** (FSS), **(F)** (VAS). CS, Corticosteroids; PRP, platelet-rich plasma; D5W, 5% dextrose in water; P, progesterone; HA, hyaluronidase; O, ozone; LA, local anesthetic; NSS, normal saline; S, splint; “+,” combine.

**FIGURE 4:**
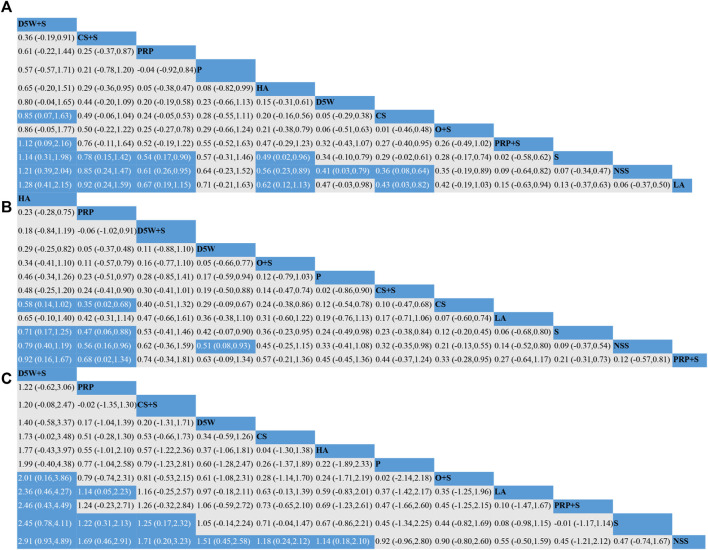
Network meta-analysis of outcomes at 3-month follow-up; **(A)** (SSS), **(B)** (FSS), **(C)** (VAS). CS, Corticosteroids; PRP, platelet-rich plasma; D5W, 5% dextrose in water; P, progesterone; HA, hyaluronidase; O, ozone; LA, local anesthetic; NSS, normal saline; S, splint; “+”, combine.

The outcome indicator involved 23 RCTs, 12 interventions, 1,458 wrists, 757 wrists in the experimental group, and 701 wrists in the control group. The consistency test and NMA were performed on the included data. The inconsistency results showed that *p* = 0.88, there was no overall inconsistency, and the nodal split method showed *p > 0*.05 between all nodes, suggesting no local inconsistency. The results of the loop inconsistency (LI) test’s inconsistency factor (IF) values ranged from 0.03 to 0.46, and the lower limit of 95% CI (confidence interval) was 0, indicating no significant inconsistency among the closed loops. Network relationships were centered on CS and NSS, forming seven trilateral closed loops (Figure 3A). The results of the NMA showed 66 two-by-two comparisons, with D5W + S outperforming CS [MD = 0.85, 95% CI (0.07,1.63)] and PRP + S [MD = 1.12, 95% CI (0.09,2.16)] with statistically significant differences; the remaining differences between local injection treatments were not significant. (Figure 4A). The final ranking of the 12 interventions using SUCRA: D5W + S [MD = 1.28, 95%CI (0.41,2.15), SUCRA = 95%] > CS + S [MD = 0.92, 95%CI (0.24,1.59), SUCRA = 82.3%] > PRP [MD = 0.67, 95%CI (0.19,1.15), SUCRA = 71.8%] > P [MD = 0.71, 95%CI (−0.21,1.63), SUCRA = 67.1%] > HA [MD = 0.62, 95%CI (0.12,1.13), SUCRA = 66.7%] > D5W [MD = 0.47, 95%CI (−0.03,0.98), SUCRA = 55.2%] > CS [MD = 0.43, 95%CI (0.03,0.82), SUCRA = 46.8%] > O + S [MD = 0.42, 95%CI (−0.19,1.03), SUCRA = 46.8%] > PRP + S [MD = 0.15, 95%CI (−0.63,0.94), SUCRA = 25.8%] > S [MD = 0.13, 95%CI (−0.37,0.63), SUCRA = 19.4%] > NSS [MD = 0.06, 95%CI (−0.37,0.50), and SUCRA = 14.7%] > LA (SUCRA = 11.4%) (Table 2; Figure 4A).

**TABLE 2:** Summary results of all SUCRA values.

SUCRA(%)							
Three months				Six months			
Treat	SSS	FSS	VAS	Treat	VAS	SSS	FSS
D5W + S	95	71.6	96.3	D5W + S	84.4	NA	NA
CS + S	82.3	48.4	74.6	CS + S	79.8	NA	NA
PRP	71.8	74.3	76.4	PRP	76.7	77.8	70.4
P	67.1	51.9	44.3	D5W	70.4	74.8	77.8
HA	66.7	89.5	53.4	HA	53.2	89.6	83.6
D5W	52.2	68.7	69.7	S	50.5	46.7	27
CS	46.8	39.4	55.7	CS	34	32.5	26.5
O + S	46.8	62.1	43.2	P	24.4	23.7	65.1
PRP + S	25.8	13.9	26.2	LA	18.9	31.9	30.9
S	19.4	26.3	23.3	NSS	7.8	23	18.7
NSS	14.7	18.4	9.1				
LA	11.4	35.5	27.7				

CS, corticosteroids; PRP, platelet-rich plasma; D5W, 5% dextrose in water; P, progesterone; HA, hyaluronidase; O, ozone; LA, local anesthetic; NSS, normal saline; S, splint; “+”, combine; ①VAS, visual analog scale; ②SSS, symptom severity score; ③FSS, functional status scale.

#### 3.3.3 Effect of functional status scale (3-month follow-up)

The outcome indicator involved 23 RCTs, 12 interventions, 1,458 wrists, 757 wrists in the experiment, and 701 wrists in the control group. The inconsistency test and NMA were performed on the included data. The inconsistency results showed that *p* = 0.88 and that there was no overall inconsistency. The nodal split method showed *p > 0*.05 between all nodes, suggesting no local inconsistency. The LI test results’ IF values ranged from 0.01 to 0.93, and the lower limit of 95% CI was from 0 to 0.35. There was inconsistency in the D5W-NSS-PRP closed loop. The reason for the inconsistency in the analysis may be that the D5W injection volume of 3 mL used in the study by Shen et al. was lower than the optimal injection volume, resulting in a lower effect size for D5W in a direct comparison of PRP and D5W (Shen et al., 2019). The network relationship was centered on CS and NSS, forming seven trilateral closed loops and one quadrilateral closed loop (Figure 3B). The results of the NMA showed that in the 66 two-by-two comparisons, HA [MD = 0.58, 95%CI (0.14,1.02)] and PRP [MD = 0.35, 95% (0.02,0.68)] were superior to CS and that NSS was lower than CS, with statistically significant differences. The remaining interventions were not significantly different from corticosteroids (Figure 4B). The final ranking of the 12 interventions using SUCRA was as follows: HA [MD = 0.92, 95%CI (0.16,1.67), SUCRA = 89.5%] > PRP [MD = 0.68, 95%CI (0.02,1.34), SUCRA = 74.3%] > D5W + S [MD = 0.74, 95%CI (−0.34,1.81), SUCRA = 71.6%] > D5W [MD = 0.63, 95%CI(-0.09,1.34), SUCRA = 68.7%] > 0 + S [MD = 0.57, 95%CI (−0.21,1.36), SUCRA = 62.1%] > P [MD = 0.45, 95%CI (−0.45.1.36), SUCRA = 51.9%] > CS + S [MD = 0.44, 95%CI (−0.37,1.24), SUCRA = 48.4%] > CS [MD = 0.33, 95%CI (−0.28,0.95), SUCRA = 39.4%] > LA [MD = 0.27, 95%CI (−0.64.1.17), SUCRA = 35.5%] > S [MD = 0.21, 95%CI (−0.31,0.73), SUCRA = 26.3%] > NSS [MD = 0.12, 95%CI (−0.57,0.81), and SUCRA = 18.4%] > PRP + S (SUCRA = 13.9%) (Table 2; Figure 4B).

#### 3.3.4 Effect of visual analog scale (3-month follow-up)

The outcome index involved 21 RCTs, 12 interventions, and 1,552 wrists, 823 in the experimental group and 729 in the control group. The inconsistency test and NMA were performed on the included data. The inconsistency results showed *p* = 0.50 and no overall inconsistency. The node split method showed *p > 0*.05 among all nodes, suggesting no local inconsistency. The LI test results’ IF values ranged from 0.43 to 2.03, and the lower limit of 95% CI was 0, indicating no significant inconsistency among the closed loops. The network relationship was centered on CS and NSS, forming five trilaterally closed loops (Figure 3C). The results of the NMA showed 66 two-by-two comparisons: There were no significant differences in the efficacy of each locally injected drug compared with corticosteroids; D5W + S was significantly more efficacious than O + S [MD = 2.01, 95% CI (0.16,3.86)] and PRP + S [MD = 2.46, 95% (0.43,4.49)] (Figure 4C). The final ranking of the 12 interventions using SUCRA was: D5W + S [MD = 2.91, 95%CI (0.93,4.89), SUCRA = 96.3%] > PRP [MD = 1.69, 95%CI (0.46,2.91), SUCRA = 76.4%] > CS + S [MD = 1.71, 95%CI (0.20,3.23), SUCRA = 74.6%] > D5W [MD = 1.51, 95%CI (0.45,2.58), SUCRA = 69.7%] > CS [MD = 1.18, 95%CI (0.24,2.12), SUCRA = 55.7%] > HA [MD = 1.14, 95%CI (0.18,2.10), SUCRA = 53.4%] > P [MD = 0.92, 95%CI (−0.96,2.80), SUCRA = 44.3%] > O + S [MD = 0.90, 95%CI (−0.80,2.60), SUCRA = 43.2%] > LA [MD = 0.55, 95% (-0.50,1.59), SUCRA = 27.7%] > PRP + S [MD = 0.45, 95%CI (−1.21,2.12), SUCRA = 25.8%] > S [MD = 0.47, 95%CI (−0.74,1.67), and SUCRA = 23.3%] > NSS (SUCRA = 9.1%) (Table 2; Figure 4C).

#### 3.3.5 Effect of symptom severity score (6-month follow-up)

**FIGURE 5:**
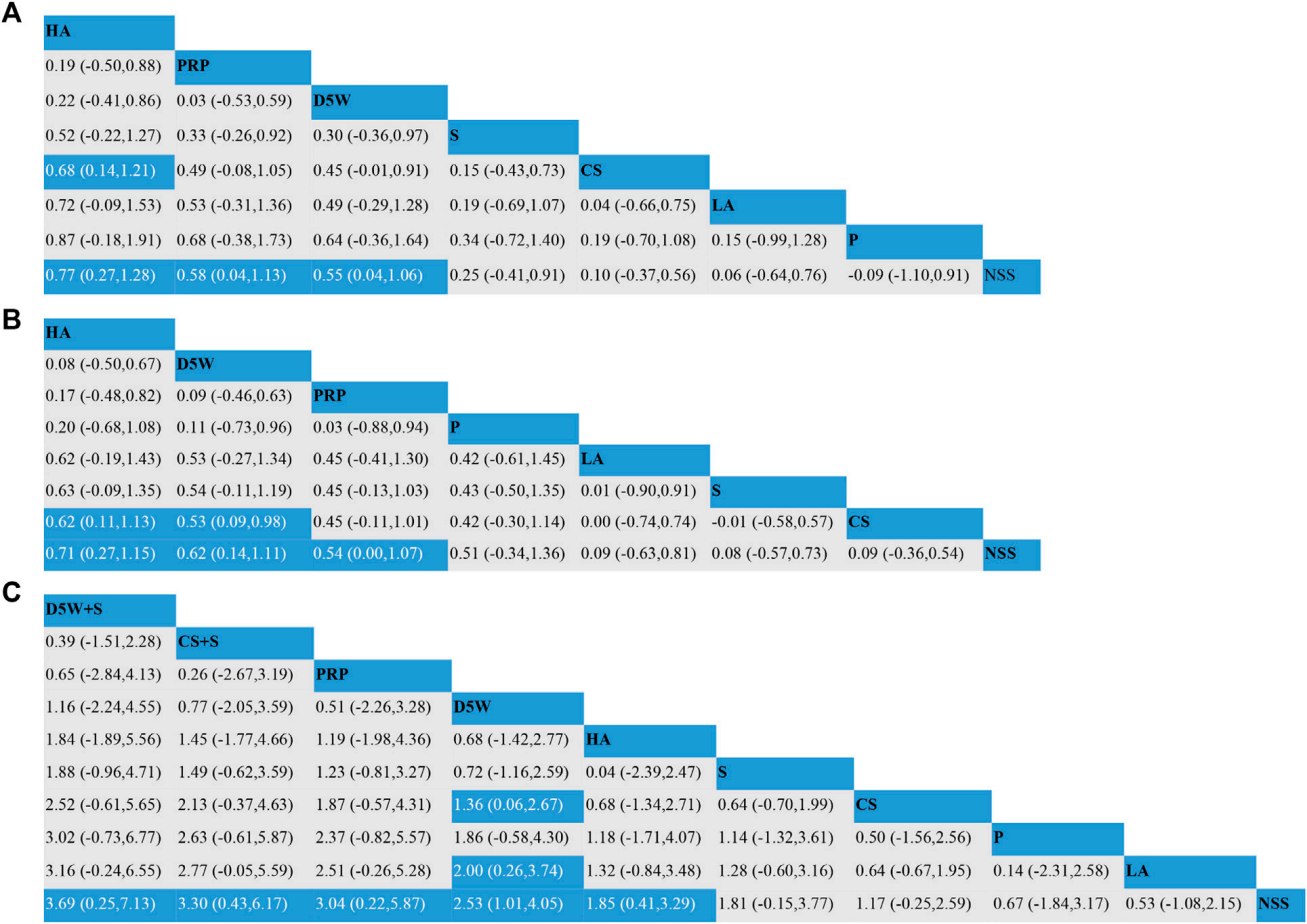
Network meta-analysis of outcomes at 6-month follow-up; **(A)** (SSS), **(B)** (FSS), **(C)** (VAS). CS, Corticosteroids; PRP, platelet-rich plasma; D5W, 5% dextrose in water; P, progesterone; HA, hyaluronidase; O, ozone; LA, local anesthetic; NSS, normal saline; S, splint; “+,” combine.

The outcome indicator involved 12 RCTs, 8 interventions, and 862 wrists, 414 in the experimental group and 448 in the control group. Inconsistency tests and NMA were performed on the included data. The inconsistency results showed *p* = 0.98 and no overall inconsistency, and the nodal split method showed *p > 0*.05 between all nodes, suggesting no local inconsistency. The LI test results’ IF values ranged from 0.01 to 0.72, and the lower limit of 95% CI was from 0 to 0.26. There was inconsistency in the CS-HA-NSS closed loop. The inconsistency in the analysis could be attributed to the fact that, unlike in most other studies, the corticosteroid used by Alsaeid et al. in the direct comparison involving CS-HA was dexamethasone, thereby leading to differences in the results of the direct and indirect comparisons (Alsaeid, 2019). The network relationship was centered on CS and NSS, and 28 pairs were compared two-by-two, forming three trilateral closed loops and two quadrilateral closed loops (Figure 3D). The results of the NMA showed that HA [MD = 0.68, 95% CI (0.14,1.21)] was significantly superior to CS, and the remaining treatment options were not significantly different compared with CS. The final ranking of the eight interventions using SUCRA was as follows: HA [MD = 0.77, 95%CI (0.27,1.28), SUCRA = 89.6%] > PRP [MD = 0.58, 95%CI (0.04,1.13), SUCRA = 77.8%] > D5W [MD = 0.55, 95%CI (0.04,1.06), SUCRA = 74.8%] > S [MD = 0.25, 95%CI (−0.41,0.91), SUCRA = 46.7%] > CS [MD = 0.10, 95%CI (−0.37,0.56), SUCRA = 32.5%] > LA [MD = 0.06, 95%CI (−0.64,0.76), SUCRA = 31.9%] > P [MD = −0.09, 95%CI (−1.10,0.91), and SUCRA = 23.7%] > NSS (SUCRA = 23%) (Table 2; Figure 5A).

#### 3.3.6 Effect of functional status scale (6-month follow-up)

The outcome indicator involved 12 RCTs, 8 interventions, and 862 wrists, 414 in the experimental group and 448 in the control group. The inconsistency test for the included data showed *p* = 0.94, no overall inconsistency, and the node split method showed *p* > 0.05 between all nodes, no local inconsistency. The IF values of the LI test results ranged from 0.01 to 0.85, and the lower 95% CI limit ranged from 0 to 0.27, Inconsistencies found in the closed loop of the D5W-NSS-PRP can be attributed to the injection volume originating from D5W (Shen et al., 2019). The network relationship was centered on CS and NSS, and 28 pairs were compared two-by-two, forming three trilateral closed loops and two quadrilateral closed loops (Figure 3E). The results of the NMA showed that HA [MD = 0.62, 95% CI (0.11,1.13)] and D5W [MD = 0.53, 95% CI (0.09,0.98)] were significantly better than CS. whereas the remaining treatment regimens were not significantly different compared with CS. The final ranking of the eight interventions was performed using SUCRA: HA [MD = 0.71, 95%CI (0.27,1.15), SUCRA = 83.6%] > D5W [MD = 0.62, 95%CI (0.14,1.11), SUCRA = 77.8%] > PRP [MD = 0.54, 95%CI (0.00,1.07), SUCRA = 70.4%] > P [MD = 0.51, 95%CI (−0.34,1.36), SUCRA = 65.1%] > LA [MD = 0.09, 95%CI (−0.63,0.81), SUCRA = 30.9%] > S [MD = 0.08, 95%CI (−0.57,0.73), SUCRA = 27%] > CS [MD = 0.09, 95% CI (−0.36,0.54), and SUCRA = 26.5%) > NSS (SUCRA = 18.7%) (Table 2; Figure 5B).

#### 3.3.7 Effect of visual analog scale (6-month follow-up)

The outcome index involved 13 RCTs, 10 interventions, and 1,109 wrists, 555 in the experimental group and 554 in the control group. The inconsistency test and reticulation meta-analysis were performed on the included data. The inconsistency result showed *p* = 0.98 and no overall inconsistency, and the node splitting method showed *p > 0*.05 among all nodes, suggesting no local inconsistency. The LI test results’ IF values ranged from 0.56 to 0.79, and the lower limit of 95% CI was 0, indicating no significant inconsistency among the closed loops. The network relationship was centered on CS and NSS, with 45 pairs compared two-by-two, forming two trilateral closed loops (Figure 3F). The results of the NMA showed that D5W [MD = 1.36, 95% CI (0.06,2.67) had better efficacy compared to CS. The final ranking of the eight interventions using SUCRA was as follows: D5W + S [MD = 3.69, 95% CI (0.25,7.13), SUCRA = (84.4%)] > CS + S [MD = 3.30, 95% CI (0.43,6.17), SUCRA = (79.8%)] > PRP [MD = 3.04, 95% CI (0.22,5.87), SUCRA = 76.7%] > D5W [MD = 2.53, 95% CI (1.01,4.05), SUCRA = 70.4%] > HA [MD = 1.85, 95% CI (0.41,3.29), SUCRA = 53.2%] > S [MD = 1.81, 95% CI (−0.14,3.77), SUCRA = 50.5%] > CS [MD = 1.17, 95% CI (−0.25,2.59), SUCRA = 34%] > P [MD = 0.67, 95% CI (−1.84,3.18), SUCRA = 24.4%] > LA [MD = 0.53, 95% CI (−1.09,2.15), and SUCRA = 18.9%] > NSS (SUCRA = 7.8%) (Table 2; Figure 5C).

### 3.4 Publication bias

**FIGURE 6:**
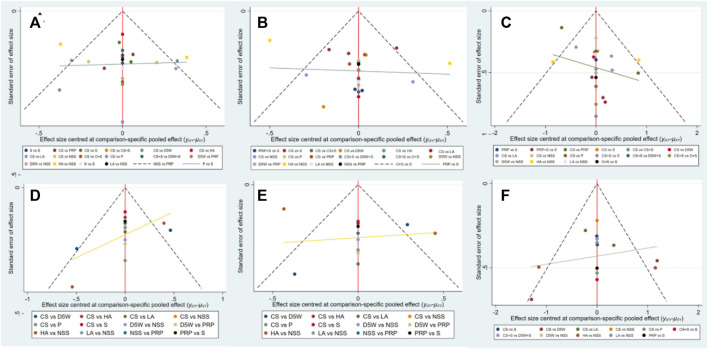
Funnel plots at 3-month follow-up, **(A)** (SSS), **(B)** (FSS), **(C)** (VAS); At 6-month follow-up, **(D)** (SSS), **(E)** (FSS), **(F)** (VAS). CS, Corticosteroids; PRP, platelet-rich plasma; D5W, 5% dextrose in water; P, progesterone; HA, hyaluronidase; O, ozone; LA, local anesthetic; NSS, normal saline; S, splint; “+,” combine.

Although the included studies were symmetrically distributed within the funnel in the funnel plots for each outcome indicator, this paper obtained data from multiple reflections from the same laboratory (wu). Therefore, publication bias may exist (Figure 6).

## 4 Discussion

CS has been commonly used as a treatment for CTS and has shown short-term efficacy. According to a systematic review that included 1,028 subjects from 10 publications, there was no significant difference in symptom severity, functional status, or pain indices between surgical treatment and topical CS injections at 3 and 12 months post-treatment (Shi et al., 2020). Although CTS release is one of the most commonly used and safest surgical interventions, complication rates range from 1% to 12% (Boeckstyns and Sørensen, 1999). The incidence of side effects with conservative treatment, such as steroid injections, is much lower than that with surgery and can provide adequate therapeutic benefit with minimal risk for a large proportion of the population (Hui et al., 2005); thus, we excluded surgical interventions.

In this study, NMA was used to compare the effects of local injections of different drugs on the symptom severity, functional status, and pain index in patients with CTS. The results of NMA showed that regarding improving SSS scores, D5W + S was the most effective at the 3-month follow-up, followed by CS + S, with HA being most effective at the 6-month follow-up, followed by PRP. Regarding improvement of FSS scores, HA ranked first among the eight interventions at 3 and 6 months, followed by PRP at 3 months and D5W at 6 months. The ranked results excluded the treatment of local injection combined with splinting because no follow-up data were available for BCTQ scores at 6 months. In improving VAS scores, D5W + S was more effective than the other treatments at both 3 and 6 months, followed by PRP at 3 months and CS + S at 6 months. However, the clinical efficacy difference was nonsignificant compared with each drug.

Our findings are consistent with those of other systematic reviews, with some differences. In a systematic review, Jiang et al. included 8 RCTs with 220 subjects. They showed that local PRP injections were superior to other conservative treatments in medium-term efficacy in relieving pain, improving wrist function and symptoms, reducing median nerve swelling, and partially improving electrophysiological indices (Jiang et al., 2022). A similar conclusion was reached in an NMA by Gao et al., which included 12 randomized controlled trials with 749 patients (817 hands). Their results showed that PRP injections were most likely to provide symptomatic relief, improve function, and relieve pain; among the injections of CS, D5W, and PRP, the PRP injections were the most recommended treatment (Gao et al., 2023). However, the aforementioned study did not include a comparison of local injections combined with splinting. If we exclude the results of the local injection therapy combined with splinting, the results obtained are consistent with those reported by GAO et al. Our results were, however, somewhat different from those obtained by Lin et al., who performed an NMA to compare the effects of CS, D5W, PRP, placebo injections, and splinting for CTS. They included 10 studies (7 randomized controlled trials and 3 prospective trials) that included 497 patients with 518 confirmed carpals. Their results suggest that D5W injections are the best option for symptomatic relief, followed by PRP injections. Splinting was more effective than PRP and D5W injections regarding functional improvement (Lin C. P. et al., 2020). The reasons for this may be due to different inclusion and exclusion criteria, the small number of studies and types of interventions included by Lin et al. (10 studies, 5 treatments), the presence of splinting in combination with local injection treatment in some studies, and that the authors did not consider it as the same treatment as a local injection alone, which may have created a high effect size for D5W. In this study, strict inclusion criteria were developed, and 26 studies were included, all of which were RCTs with high-quality literature (Raeissadat et al., 2018; Güven et al., 2019). In addition, for the use of local injection therapy combined with splinting, we treated this as a separate intervention, separate from injection therapy alone and splinting alone, to avoid confounding factors. Further comparisons of long-term (6 months) outcomes were made. However, deficiencies remain. Recent systematic reviews on regenerative injections for CTS have mainly involved comparisons of positioning modalities and injected drugs. Yang et al. compared the effect of different positioning modalities on the efficacy of local CS injections; they found that ultrasound-guided injections were superior to landmark-guided injections on the Boston Carpal Tunnel Syndrome Questionnaire, SSS, and Functional Status Scale (Yang et al., 2021). The same results were obtained in a recently published meta-analysis (Alhindi et al., 2023). However, because of the large number of interventions included in this study and the small amount of literature corresponding to each intervention, performing a subgroup analysis for whether ultrasound was used was impossible; 13 of the 26 RCTs used ultrasound localization, mainly involving PRP, D5W, and HA.

By contrast, ultrasound localization was mostly not used for CS injections, possibly resulting in high effect sizes for PRP, D5W, and HA. Babaei-Ghazani et al. found that the improvement in symptom severity, functional impairment, and pain index scores of CS for CTS was at its largest effect size at the 6-week follow-up (Babaei-Ghazani et al., 2022). Therefore some of the previous studies have less than 3 months of follow-up, which may result in a high effect size for corticosteroids.

The pathophysiological mechanism of CTS remains unclear. The increased pressure in the carpal tunnel may be one of the mechanisms due to the fibrosis of the connective tissue of the median nerve and the sub-synovial region, increased carpal tunnel contents, and adhesions of the synovial connective tissue of the flexor tendons surrounding the median nerve (Elawamy et al., 2020). The experimental evidence shows that inflammation is also the cause of CTS in most patients (Chen et al., 2015). According to a meta-analysis, local injection therapy may work through two aspects: the drug’s action and hydrodynamic separation (Buntragulpoontawee et al., 2020). Smith et al. described ultrasound-guided injections of lidocaine and CS in patients with CTS, and the concept of nerve hydro dissection was proposed (Smith et al., 2008). Hydro dissection can release adhesions, liberating the nerve from the surrounding tissue (Aboonq, 2015; Bland, 2018). Evers et al. demonstrated that injection of NSS into the carpal tunnel alone reduced the longitudinal gliding resistance of the median nerve and reduced nerve compression (Evers et al., 2018).

Studies have found that PRP is rich in growth factors that promote nerve repair and regeneration, namely, platelet-derived growth factor, transforming growth factor-beta, insulin-like growth factor, and vascular endothelial growth factor, these cytokines promote wound healing, reduce inflammation to repair the damaged median nerve, and increase the production of alpha-2 collagen and type III collagen in flexor support band cells, reducing the pressure in the carpal tunnel (Allampallam et al., 2000; Sampson et al., 2008; Picard et al., 2015). However, there are no uniform standards for the preparation process of PRP. The composition of the preparation is affected by individual body composition, and the optimal concentration of platelets is uncertain; in addition, studies have shown that the optimal concentration of PRP is from four to seven times and that anything less than four times or more than eight times is ineffective or inhibits the healing process (Jiang et al., 2022).

HA is an enzyme that catalyzes the hydrolysis of hyaluronic acid and promotes the rebuilding of the nerve myelin sheath by degrading the accumulated hyaluronic acid. HA also reduces the inflammatory response, reduces viscoelasticity, increases tissue permeability, and allows local anesthetic to diffuse through the surrounding tissue (Dunn et al., 2010; Elawamy et al., 2020). In a randomized controlled trial compared the effect of ultrasound-guided HA with that of dexamethasone on hydro dissection in mild to moderate CTS; they showed that the improvement in symptoms, function, electrophysiological performance, and cross-sectional area of the median nerve (CSA) in MN was significantly greater in the HA group than in the dexamethasone group at the 6-month follow-up (Alsaeid, 2019). Another study reported that ultrasound-guided HA injections combined with NSS resulted in significant improvements in pain, function, electro-physiological parameters and CSA of MN in patients with mild to moderate CTS at a 6-month follow-up compared with NSS injections alone (Elawamy et al., 2020). Moreover, the efficacy of HA is related to the volume injected, and a recent randomized controlled trial comparing HA injection alone with NSS injection alone showed different results. Su et al. assessed 32 patients (17 in the HA group and 15 in the control group) at the 6-month follow-up and they found non-significant differences between the HA and control groups, with the exception of BCTQ and number analog scoring at week 2 post-injection (Su et al., 2021). Unlike in the studies conducted by Elawamy (10 mL) and Alsaeid (5 mL), Su et al. used 2.5 mL, which might not achieve the effect of aqueous dissection. Thus, the mechanisms of HA in the treatment of CTS may be associated with the effect of aqueous dissection.

The D5W is often used for microinjection because it has an osmotic pressure similar to that of saline and can inhibit the activation of capsaicin-sensitive receptors (e.g., transient receptor potential vanilloid receptor-1), blocking the release of substance P and calcitonin gene-related peptide and relieving neurogenic inflammatory pain (Li et al., 2021). Several studies have shown that D5W is more effective than CS and NSS in reducing pain and disability, improving electrophysiological response parameters and reducing CSA (Wu et al., 2017b; Wu et al., 2018; Shen et al., 2019). However, in Babaei-Ghazani, D5W had similar efficacy to CS in improving pain intensity, functional limitations in daily life, electrophysiological parameters, and ultrasound findings, with no significant difference between the two during the 3-month follow-up period (Babaei-Ghazani et al., 2022). The major difference between the two experiments was the volume of CS: Babaei-Ghazani used 1 mL 40 mg/mL tretinoin, and Wu used 3 mL 10 mg/mL of tretinoin combined with 2 mL NSS. One study reported that CS combined with NSS does not produce a hydrodynamic stripping effect but may reduce trimethoprim concentration and affect the efficacy of the treatment (Wang et al., 2021). It can be established that a local injection of 5 mL D5W is at least as effective as CS, and this finding is consistent with those of this paper.

Progesterone has anti-inflammatory effects and is essential in local inflammatory healing, myelin repair, and axonal regeneration (Bahrami et al., 2015). In two studies by Raeissadat and Bahrami et al., which included 78 (41 in the progesterone group and 37 in the corticosteroid group) and 54 (24 in the progesterone group and 30 in the CS group) women, respectively, the progesterone group was comparable to the CS group in efficacy at the 3-month follow-up, and at the 6-month follow-up, the progesterone group was significantly better than the CS group (Bahrami et al., 2015; Raeissadat et al., 2017). However, these studies had obvious limitations in that they were performed exclusively on female patients.

The neutral splint is the first-line treatment option for CTS and is widely used in clinical practice (Currie et al., 2022). Considerable evidence shows that long-term use of a neutral splint effectively improves the functional status of CTS. In Karina et al., 24 weeks of night rest splinting reduced the probability of patients taking surgical treatment significantly, with a 59% and 80% conversion rate in the experimental group and control group, respectively, as well as benefits in satisfaction, symptom severity, and functional limitations (Lewis et al., 2020). According to a recent systematic review in Cochanrce, whether splints should be worn full-time or only at night and whether long-term use is preferable to short-term use remain unclear. However, low-quality evidence suggests that benefits will be realized with long-term use (Karjalainen et al., 2023). Therefore, local injections combined with splinting may contribute to improved results. In this paper, local injection therapy combined with splinting was also analyzed. The results showed that the D5W combined splinting group and the CS combined splinting group improved the severity of symptoms, functional status, and pain index better than local injection alone or splinting alone. However, PRP combined with splinting was not superior to PRP injections alone and was not even significantly different from splinting therapy alone, and the following factors may be present.

1. Fewer studies were included. There was only one study involving PRP combined with splinting, and the sample size was small; thus, bias may have occurred.
2. Raeissadat et al. did not use ultrasound localization and the PRP dose was 1 mL, which was lower than other experiments in which PRP was injected alone, resulting in inadequate efficacy. (Raeissadat et al., 2018).
3. The follow-up time was only 10 weeks (Raeissadat et al., 2018), which is less than that in other studies, and a delayed effect of PRP was reported in the relevant literature (Wu et al., 2017a; Gado and El-Banna, 2020).

And in a related study, which confirms our speculation, compared to the study of Raeissadat et al., Gado et al. used ultrasound-guided injection using an in-plane ulnar approach; the puncture needle was passed from the ulnar side of the wrist joint to the MN, and 2 mL of PRP was injected, and the flexor supporting band of nerves was stripped with water, and the remaining 1 mL of PRP was injected underneath the MN, and evaluated the results of the treatment in 40 patients (PRP + S group 20 patients and splint group 20 patients) at 3-month follow-up, and their results showed that the differences in VAS, SSS, FSS and CSA between the two groups at 1 month after treatment were not statistically significant; however, patients in the PRP combined with brace group showed better improvement in all of the above indexes than those in the brace-only group at 3 months after treatment (Gado and El-Banna, 2020), indicating that there is a delayed effect of PRP and that PRP injection dose of 3 mL is better than 1 mL.

Additionally, the volume of injection used for PRP in the literature mostly ranged from 1 to 3.5. We found that the optimal volume of injection for PRP may be approximately 2 mL by comparing the change in BCTQ and VAS scores for different volumes of PRP; the optimal volume of NSS for aqueous autopsy ranged from 2.5 to 3.5 mL using the same method. Lin et al. found that 4 mL 5% D5W was superior to 1 mL and 2 mL D5W in symptom relief and functional improvement in CTS at weeks 1, 4, and 12 post-injection by comparing the efficacy of ultrasound-guided injections of different doses of D5W in CTS (Lin M. T. et al., 2020). By contrast, most of the studies on D5W included in this paper used 5 mL, and it is therefore uncertain as to whether an injection volume higher than 5 mL would provide better results. The same conclusion was reached in a systematic review by Lam et al., and CS was found to be superior to lower doses in the high-dose group (Lam et al., 2023). Further research should directly compare different injection volumes of PRP, D5W, and CS to determine their optimal dose.

### 4.1 Limitations

This study has limitations. The water separators applied are mainly lidocaine and saline; thus, the use of lidocaine or saline in the various studies may also affect the experimental results. High-quality evidence has not been found on whether ultrasound localization is superior to landmark localization. Different types of corticosteroids may have different efficacy, and three types of corticosteroids were used in the included studies. However, they are treated as the same intervention in this paper. In addition, despite some advantages of PRP in the treatment of CTS, there are currently no uniform standards for the preparation of PRP, resulting in different studies using different PRP components. For example, both platelet concentration and leukocyte content can be affected by different preparation methods as well as the patient’s own body composition. These can all have an impact on the efficacy of PRP. However, no sub-group analysis was performed owing to the small number of studies reviewed in this meta-analysis.

## 5 Conclusion

In summary, the current SUCRA ranking, which considers all indicators, shows that D5W + S is the best treatment option, followed by PRP, HA, CS + S, D5W, P, 0 + S, CS, S, LA, PRP + S, and NSS. The PRP, HA, and D5W + S treatments were more effective than corticosteroids in improving function, symptoms, and pain in the short term (3 months) and the long term (6 months). No severe complications were reported in the included literature, and the safety profile was good. It is hoped that future studies will further determine the optimal dose of different local injection treatments, reach a consensus regarding the method of PRP preparation, and conduct longer (>6 months) follow-ups.

## Data Availability

The original contributions presented in the study are included in the article/Supplementary Material, further inquiries can be directed to the corresponding author.

## Abbreviations

BCTQ, Boston Carpal Tunnel Questionnaire; CI, Confidence interval; CS, Corticosteroid; CSA, Cross-sectional area of the median nerve; CTS, Carpal tunnel syndrome; D5W, 5% dextrose in water; FSS, Functional severity scale; HA, Hyaluronidase; IF, Inconsistency factor; LA, Local anesthetics; LI, Loop inconsistency; MD, Mean difference; MeSH, Medical subject headings; MN, Median nerve; NMA, Network meta-analysis; NSS, Normal saline; P, Progesterone; PRP, Platelet-rich plasma; RCT, Randomized controlled trial; SSS, Symptom severity scale; SUCRA, Surface under the cumulative ranking curve; US, Ultrasound; VAS, Visual analog score.
