# Supplementary material for: Local injection therapy for carpal tunnel syndrome: a network meta-analysis of randomized controlled trial
Source: Front Pharmacol. 2023 Aug 24;14:1140410. doi: 10.3389/fphar.2023.1140410 (PMC10484596; doi:10.3389/fphar.2023.1140410)
Supplement: Supplementary file 1 [file DataSheet1.pdf]

Supplementary Material

Local injection therapy for carpal tunnel syndrome: A network meta-analysis of randomized controlled trial

TianQi Zhou[1], ShuangChun Ai[2*], ZhuoRao Wu[1],XingYun Gou[1], HaiSha Xia[1], JiLin Ding[2]

*** Correspondence:** ShuangChun Ai, Chief Physician, Master Instructor, Director of the Department of Rehabilitation. email:aishuangchun@163.com

1 Supplementary Table 1

search strategy

Pubmed

#1	((((((("Carpal Tunnel Syndrome"[Mesh]) OR (Carpal Tunnel Syndromes[Title/Abstract])) OR (Syndrome, Carpal Tunnel[Title/Abstract])) OR (Syndromes, Carpal Tunnel[Title/Abstract])) OR (Amyotrophy, Thenar, Of Carpal Origin[Title/Abstract])) OR (Median Neuropathy, Carpal Tunnel[Title/Abstract])) OR (Compression Neuropathy, Carpal Tunnel[Title/Abstract])) OR (Entrapment Neuropathy, Carpal Tunnel[Title/Abstract])) OR (Carpal Tunnel Syndrome[Title/Abstract])
#2	((((((((((((((((("Splints"[Mesh]) OR (Splint[Title/Abstract])) OR (Static Splints[Title/Abstract])) OR (Splint, Static[Title/Abstract])) OR (Splints, Static[Title/Abstract])) OR (Static Orthoses[Title/Abstract])) OR (Static Orthose[Title/Abstract])) OR (Static Splinting[Title/Abstract])) OR (Splinting, Static[Title/Abstract])) OR (Static Splint[Title/Abstract])) OR (Dynamic Splints[Title/Abstract])) OR (Splint, Dynamic[Title/Abstract])) OR (Splints, Dynamic[Title/Abstract])) OR (Dynamic Orthoses[Title/Abstract])) OR (Dynamic Splint[Title/Abstract])) OR (Dynamic Splinting[Title/Abstract])) OR (Dynamic Splintings[Title/Abstract])) OR (Splinting, Dynamic[Title/Abstract])) OR (Splints[Title/Abstract])

#3	(((((("Anesthesia, Local"[Mesh]) OR (Local Anesthesia[Title/Abstract])) OR (Anesthesia, Local[Title/Abstract])) OR (Anesthesia, Infiltration[Title/Abstract])) OR (Infiltration Anesthesia[Title/Abstract])) OR (Neural Therapy of Huneke[Title/Abstract])) OR (Huneke Neural Therapy[Title/Abstract])
#4	((((("Progesterone"[Mesh]) OR (Progesterone[Title/Abstract])) OR (Pregnenedione[Title/Abstract])) OR (Progesterone, (17 alpha)-Isomer[Title/Abstract])) OR (Progesterone, (9 beta,10 alpha)-Isomer[Title/Abstract])) OR (Progesterone, (13 alpha,17 alpha)-(+-)-Isomer[Title/Abstract])
#5	("Platelet-Rich Plasma"[Mesh]) OR (Plasma, Platelet-Rich[Title/Abstract]) OR (Platelet Rich Plasma[Title/Abstract] OR Platelet-Rich Plasma[Title/Abstract]
#6	((((((((((((("Glucose"[Mesh]) OR (Glucose[Title/Abstract])) OR (D-Glucose[Title/Abstract])) OR (D Glucose[Title/Abstract])) OR (Dextrose[Title/Abstract])) OR (Glucose, (alpha-D)-Isomer[Title/Abstract])) OR (Anhydrous Dextrose[Title/Abstract])) OR (Dextrose, Anhydrous[Title/Abstract])) OR (Glucose, (DL)-Isomer[Title/Abstract])) OR (Glucose, (L)-Isomer[Title/Abstract])) OR (L-Glucose[Title/Abstract])) OR (L Glucose[Title/Abstract])) OR (Glucose Monohydrate[Title/Abstract])) OR (Monohydrate, Glucose[Title/Abstract])) OR (Glucose, (beta-D)-Isomer[Title/Abstract])
#7	(((((("Saline Solution"[Mesh]) OR (0.9% Saline[Title/Abstract])) OR (Saline, 0.9%[Title/Abstract])) OR (0.9% NaCl[Title/Abstract])) OR (Normal Saline[Title/Abstract])) OR (Saline, Normal[Title/Abstract])) OR (Saline Solution[Title/Abstract])
#8	((((((((((((("Hyaluronic Acid"[Mesh]) OR (Acid, Hyaluronic[Title/Abstract])) OR (Amo Vitrax[Title/Abstract])) OR (Vitrax, Amo[Title/Abstract])) OR (Biolon[Title/Abstract])) OR (Etamucine[Title/Abstract])) OR (Hyaluronic Acid[Title/Abstract])) OR (Hyaluronan[Title/Abstract])) OR (Hyvisc[Title/Abstract])) OR (Luronit[Title/Abstract])) OR (Sodium Hyaluronate[Title/Abstract])) OR (Hyaluronate, Sodium[Title/Abstract])) OR (Hyaluronate Sodium[Title/Abstract])) OR (Amvisc[Title/Abstract])) OR (Healon[Title/Abstract])
#9	(((((((((("Ozone"[Mesh]) OR (Tropospheric Ozone[Title/Abstract])) OR (Ozone[Title/Abstract])) OR (Ozone, Tropospheric[Title/Abstract])) OR (Low Level Ozone[Title/Abstract])) OR (Level Ozone, Low[Title/Abstract])) OR (Ozone, Low Level[Title/Abstract])) OR (Ground Level Ozone[Title/Abstract])) OR (Level Ozone, Ground[Title/Abstract])) OR (Ozone, Ground Level[Title/Abstract])) OR (Ozone[Title/Abstract])
#10	(((("Steroids"[Mesh]) OR (Steroid[Title/Abstract])) OR (Steroids[Title/Abstract])) OR (Catatoxic Steroids[Title/Abstract])) OR (Steroids, Catatoxic[Title/Abstract])
#11	#1 AND (#2 OR #3 OR #4 OR #5 OR #6 OR #7 OR #8 OR #9 OR #10)
#12	(clinical[tiab] AND trial[tiab]) OR "clinical trials as topic"[mesh] OR "clinical trial"[pt] OR random*[tiab] OR "random allocation"[mesh]

#13	#11 AND #12

Web Of Science

((TS=(Anesthesia, Local) OR (AB=(Local Anesthesia OR Anesthesia, Local OR Anesthesia, Infiltration OR Infiltration Anesthesia OR Neural Therapy of Huneke OR Huneke Neural Therapy))) OR (TS=(Ozone) OR (AB=(Tropospheric Ozone OR Ozone OR Ozone, Tropospheric OR Low Level Ozone OR Level Ozone, Low OR Ozone, Low Level OR Ground Level Ozone OR Level Ozone, Ground OR Ozone, Ground Level OR Ozone))) OR (TS=("Splints") OR (AB=(Splint OR Static Splints OR Splint, Static OR Splints, Static OR Static Orthoses OR Static Orthose OR Static Splinting OR Splinting, Static OR Static Splint OR Dynamic Splints OR Splint, Dynamic OR Splints, Dynamic OR Dynamic Orthoses OR Dynamic Splint OR Dynamic Splinting OR Dynamic Splintings OR Splinting, Dynamic OR Splints))) OR (TS=(Steroids) OR (AB=(Steroid OR Catatoxic Steroids OR Steroids, Catatoxic))) OR (TS=(Hyaluronic Acid) OR (AB=(Acid, Hyaluronic OR Amo Vitrax OR Vitrax, Amo OR Biolon OR Etamucine OR Hyaluronic Acid OR Hyaluronan OR Hyvisc OR Luronit OR Sodium Hyaluronate OR Hyaluronate, Sodium OR Hyaluronate Sodium OR Amvisc OR Healon))) OR (TS=(Saline Solution) OR (AB=(0.9% Saline OR Saline, 0.9% OR 0.9% NaCl OR Normal Saline OR Saline, Normal OR Saline Solution))) OR (TS=(Glucose) OR (AB=(Glucose OR D-Glucose OR D Glucose OR Dextrose OR Glucose, alpha-D-Isomer OR Anhydrous Dextrose OR Dextrose, Anhydrous OR Glucose, DL-Isomer OR Glucose, L-Isomer OR L-Glucose OR L Glucose OR Glucose Monohydrate OR Monohydrate, Glucose OR Glucose, beta-D-Isomer))) OR (TS=(Progesterone) OR (AB=(Progesterone OR Pregnenedione OR Progesterone, 17 alpha-Isomer OR Progesterone, 9 beta,10 alpha-Isomer OR Progesterone, 13 alpha,17 alpha-+--Isomer))) OR (TS=(Platelet-Rich Plasma) OR (AB=(Plasma, Platelet-Rich OR Platelet Rich Plasma OR Platelet-Rich Plasma)))) AND (TS=(Carpal Tunnel Syndrome) OR (AB=(Carpal Tunnel Syndromes OR Syndrome, Carpal Tunnel OR Syndromes, Carpal Tunnel OR Amyotrophy, Thenar, Of Carpal Origin OR Median Neuropathy, Carpal Tunnel OR Compression Neuropathy, Carpal Tunnel OR Entrapment Neuropathy, Carpal Tunnel OR Carpal Tunnel Syndrome))) AND (TS=(Clinical Trials as Topic) OR (AB=(Clinical OR trial OR clinical trial OR random OR randomized controlled trial OR controlled clinical trial OR randomized OR randomly)))

Embase

#13	#1 AND #11 AND #12
#12	#2 OR #3 OR #4 OR #5 OR #6 OR #7 OR #8 OR #9 OR #10
#11	'clinical trial' OR 'random' OR 'randomized controlled trial' OR 'controlled clinical trial' OR 'randomized' OR 'randomly':ti,ab
#10	'progesterone' OR 'pregnenedione' OR 'progesterone, 17 alpha-isomer' OR 'progesterone, 9 beta,10 alpha-isomer' OR 'progesterone, 13 alpha,17 alpha-+--isomer':ti,ab

#9	'local anesthesia' OR 'anesthesia, local' OR 'anesthesia, infiltration' OR 'infiltration anesthesia' OR 'neural therapy of huneke' OR 'huneke neural therapy':ti,ab
#8	'splint' OR 'static splints' OR 'splint, static' OR 'splints, static' OR 'static orthoses' OR 'static orthose' OR 'static splinting' OR 'splinting, static' OR 'static splint' OR 'dynamic splints' OR 'splint, dynamic' OR 'splints, dynamic' OR 'dynamic orthoses' OR 'dynamic splint' OR 'dynamic splinting' OR 'dynamic splintings' OR 'splinting, dynamic or splints':ti,ab
#7	'plasma, platelet-rich' OR 'platelet rich plasma' OR 'platelet-rich plasma':ti,ab
#6	'glucose' OR 'd-glucose' OR 'd glucose' OR 'dextrose' OR 'glucose, alpha-d-isomer' OR 'anhydrous dextrose' OR 'dextrose, anhydrous' OR 'glucose, dl-isomer' OR 'glucose, l-isomer' OR 'l-glucose' OR 'l glucose' OR 'glucose monohydrate' OR 'monohydrate, glucose' OR 'glucose, beta-d-isomer':ti,ab
#5	'0.9% saline' OR 'saline, 0.9%' OR '0.9% nacl' OR 'normal saline' OR 'saline, normal' OR 'saline solution':ti,ab
#4	'acid, hyaluronic' OR 'amo vitrax' OR 'vitrax, amo' OR 'biolon or etamucine' OR 'hyaluronic acid' OR 'hyaluronan' OR 'hyvisc' OR 'luronit' OR 'sodium hyaluronate' OR 'hyaluronate, sodium' OR 'hyaluronate sodium' OR 'amvisc' OR 'healon':ti,ab
#3	'steroid' OR 'catatoxic steroids' OR 'steroids, catatoxic' OR 'steroids':ti,ab
#2	'tropospheric ozone' OR 'ozone' OR 'ozone, tropospheric' OR 'low level ozone' OR 'level ozone, low' OR 'ozone, low level' OR 'ground level ozone' OR 'level ozone, ground' OR 'ozone, ground level' OR 'ozone':ti,ab
#1	'carpal tunnel syndrome'/exp OR 'carpal tunnel syndrome' OR 'carpal tunnel syndromes' OR 'syndrome, carpal tunnel' OR 'syndromes, carpal tunnel' OR 'amyotrophy, thenar, of carpal origin' OR 'median neuropathy, carpal tunnel' OR 'compression neuropathy, carpal tunnel' OR 'entrapment neuropathy, carpal tunnel' OR 'carpal tunnel syndrome':ti,ab

Cochrane

#1	MeSH descriptor: [Carpal Tunnel Syndrome] explode all trees
#2	(Amyotrophy, Thenar, Of Carpal Origin OR Syndromes, Carpal Tunnel OR Compression Neuropathy, Carpal Tunnel OR Median Neuropathy, Carpal Tunnel OR Syndrome, Carpal Tunnel OR Entrapment Neuropathy, Carpal Tunnel OR Carpal Tunnel Syndromes):ti,ab,kw

#3	#1 OR #2
#4	MeSH descriptor: [Clinical Trials as Topic] explode all trees
#5	(Clinical OR trial OR clinical trial OR random OR randomized controlled trial OR controlled clinical trial OR randomized OR randomly):ti,ab,kw
#6	#4 OR #5
#7	MeSH descriptor: [Ozone] explode all trees
#8	(Ozone OR Ground Level Ozone OR Ozone, Ground Level OR Level Ozone, Ground OR Level Ozone, Low OR Ozone, Low Level OR Tropospheric Ozone OR Ozone, Tropospheric OR Low Level Ozone):ti,ab,kw
#9	#7 OR #8
#10	MeSH descriptor: [Steroids] explode all trees
#11	(Steroids OR Steroid OR Catatoxic Steroids OR Steroids, Catatoxic):ti,ab,kw
#12	#10 OR #11
#13	MeSH descriptor: [Hyaluronic Acid] explode all trees
#14	(Amvisc OR Hyaluronate Sodium OR Sodium Hyaluronate OR Hyaluronate, Sodium OR Hyaluronan OR Amo Vitrax OR Vitrax, Amo OR Etamucine OR Hyvisc OR Luronit OR Acid, Hyaluronic OR Biolon OR Healon):ti,ab,kw
#15	#13 OR #14
#16	MeSH descriptor: [Saline Solution] explode all trees
#17	(0.9% Saline OR Saline, 0.9% OR 0.9% NaCl OR Normal Saline OR Saline, Normal OR Saline Solution):ti,ab,kw
#18	#16 OR #17
#19	MeSH descriptor: [Glucose] explode all trees
#20	(Glucose OR D-Glucose OR D Glucose OR Dextrose OR Glucose, alpha-D-Isomer OR Anhydrous Dextrose OR Dextrose, Anhydrous OR Glucose, DL-Isomer OR Glucose, L-Isomer OR L-Glucose OR L Glucose OR Glucose Monohydrate OR Monohydrate, Glucose OR

	Glucose, beta-D-Isomer):ti,ab,kw
#21	#19 OR #20
#22	MeSH descriptor: [Platelet-Rich Plasma] explode all trees
#23	(Plasma, Platelet-Rich OR Platelet Rich Plasma OR Platelet-Rich Plasma):ti,ab,kw
#24	#22 OR #23
#25	MeSH descriptor: [Anesthesia, Local] explode all trees
#26	(Local Anesthesia OR Anesthesia, Local OR Anesthesia, Infiltration OR Infiltration Anesthesia OR Neural Therapy of Huneke OR Huneke Neural Therapy):ti,ab,kw
#27	#25 OR #26
#28	MeSH descriptor: [Splints] explode all trees
#29	(Splint OR Static Splints OR Splint, Static OR Splints, Static OR Static Orthoses OR Static Orthose OR Static Splinting OR Splinting, Static OR Static Splint OR Dynamic Splints OR Splint, Dynamic OR Splints, Dynamic OR Dynamic Orthoses OR Dynamic Splint OR Dynamic Splinting OR Dynamic Splintings OR Splinting, Dynamic OR Splints):ti,ab,kw
#30	#28 OR #29
#31	MeSH descriptor: [Splints] explode all trees
#32	(Progesterone OR Pregnenedione OR Progesterone, 17 alpha-Isomer OR Progesterone, 9 beta,10 alpha-Isomer OR Progesterone, 13 alpha,17 alphaIsomer):ti,ab,kw
#33	#31 OR #32
#34	#9 OR #12 OR #15 OR #18 OR #21 OR #24 OR #27 OR #30 OR #33
#35	#3 AND #6 AND #34
